# Berberine Inhibits FOXM1 Dependent Transcriptional Regulation of POLE2 and Interferes With the Survival of Lung Adenocarcinoma

**DOI:** 10.3389/fphar.2021.775514

**Published:** 2022-01-31

**Authors:** Lulu Ni, Ping Sun, Xiaochun Fan, Zhongjie Li, Hongli Ren, Jiangan Li

**Affiliations:** ^1^ Department of Basic Medicine, Jiangnan University, Wuxi, China; ^2^ Department of Pathology, The Affiliated Wuxi NO. 2 People’s Hospital of Nanjing Medical University, Wuxi, China; ^3^ Department of Emergency, The Affiliated Wuxi NO. 2 People’s Hospital of Nanjing Medical University, Wuxi, China; ^4^ Institute of Science, Technology and Humanities, Shanghai University of Traditional Chinese Medicine, Shanghai, China

**Keywords:** berberine, FOXM1, POLE2, DNA replication, lung adenocarcinoma, survival

## Abstract

**Background:** Berberine is one of the most interesting and promising natural anticancer drugs. POLE2 is involved in many cellular functions such as DNA replication and is highly expressed in a variety of cancers. However, the specific molecular mechanism of berberine interfering with POLE2 expression in lung adenocarcinoma (LUAD) is still unknown to a great extent.

**Method:** The KEGG database (Release 91.0) and Gene Ontology (GO) category database were used for functional annotation of differentially expressed genes after berberine treatment. Reproducibility assessment using TCGA dataset. The biological functions of berberine in LUAD were investigated by a series of *in vitro* and *in vivo* experiments: MTT, colony formation, mouse xenograft and plasmid transfection. The molecular mechanisms of berberine were demonstrated by plasmid transfection, quantitative RT-PCR and Western blotting.

**Result:** The elevated expression of FOXM1 and the high enrichment of DNA replication pathway were confirmed in LUAD by microarray and TCGA analysis, and were positively correlated with poor prognosis. Functionally, berberine inhibited the proliferation and survival of LUAD cell lines *in vitro* and *in vivo*. Mechanistically, berberine treatment down regulated the expression of FOXM1which closely related to survival, survival related genes in Cell cycle and DNA replication pathway, and significantly down regulated the expression of survival related POLE2. Interestingly, we found that the transcription factor FOXM1 could act as a bridge between berberine and POLE2.

**Conclusion:** Berberine significantly inhibited LUAD progression via the FOXM1/POLE2, and FOXM1/POLE2 may act as a clinical prognostic factor and a therapeutic target for LUAD. Berberine may be used as a promising therapeutic candidate for LUAD patients.

## Introduction

Lung cancer is the malignant tumor with the highest incidence and mortality in the world, and 85% of cases are NSCLC (Torre et al., 2015). Lung adenocarcinoma is the most common subtype of NSCLC, and the five-year overall survival rate is less than 18% (Miller et al., 2019). The initiation of DNA replication is the basic process of cell proliferation, and malignant proliferation is the basic feature of lung cancer cells. It is well known that the occurrence of lung adenocarcinoma is the result of imbalance of tumor suppressor genes or oncogenes, which will lead to uncontrolled proliferation of lung adenocarcinoma cells. In clinical, many mature chemotherapeutic drugs were designed to directly or indirectly inhibit DNA synthesis. Targeted DNA therapy was usually effective for the proliferation and survival of lung adenocarcinoma cells, such as cisplatin and carboplatin. They cross-linked purine bases in DNA, prevented DNA replication and repair of lung adenocarcinoma cells and promoted cell death. However, the side effects of these targeted drugs such as gastrointestinal, nephrotoxicity, ototoxicity and infection were very obvious. Therefore, it is very important and urgent to find high-efficiency and low toxicity anti lung adenocarcinoma drugs.

Plant medicine is a rich source of new pharmacological active agents against diseases. Berberine is a low toxic natural plant alkaloid with broad spectrum of biological and pharmacological activities, including anti diabetes (Wang et al., 2019), antidiarrheal (Joshi et al., 2011), anti-cancer (Liu et al., 2019) and antibacterial (Peng et al., 2015). At present, berberine has been evaluated in many clinical trials. Berberine has low toxicity to healthy cells and high cytotoxicity to cancer cells, which makes its research in anticancer become the most promising. Since the first study on the cytotoxicity in cancer cells of berberine in 1986 (Wang et al., 1986), its anticancer properties have been proved in many cancer cell studies (Kettmann et al., 2004; Yu et al., 2007; Pazhang et al., 2011; Zheng et al., 2014; Och et al., 2019), which makes berberine one of the most interesting and promising natural anticancer drugs.

Although many studies had shown that berberine against lung adenocarcinoma mainly focused on different cell signaling pathways, including Sin3A/TOP2B (Chen et al., 2020), Bcl-2/Bax (Li et al., 2018), mTOR (Kumar et al., 2020) and NF-κB/COX-2, Akt/ERK (Lu et al., 2016), miR-19a/TF/MAPK (Chen et al., 2019), etc. It was not clear whether berberine could exert anticancer effect by interfering with DNA replication, the basic process of lung adenocarcinoma cell proliferation. In this study, we provided evidence that berberine inhibited DNA replication levels in lung adenocarcinoma cells and Lewis tumor xenograft mice. Berberine inhibited the proliferation of lung adenocarcinoma cells by interfering with the expression of POLE2 involved in DNA replication mediated by transcription factor FOXM1. Our study analyzed the mechanism of berberine inhibiting the proliferation of lung adenocarcinoma by using gene chip technology and TCGA, and correlated the changes of genome and transcriptome with cancer cell proliferation, DNA replication and overall survival.

## Materials and Methods

### Experiment Reagents

Berberine were purchased from Shanghai Tongtian Biotechnology Co., Ltd. (shanghai, China). Berberine were dissolved in DMSO and stored at −20°C. RPMI 1640 and Dulbeberberineo’s modified eagle medium (DMEM) for culture were purchased from Gibco (Grand Island, NY, United States); fetal bovine serum (FBS) was from HyClone (South Logan, United States) and trypsin was from Gibco (Grand Island, NY, United States). Thiazoles [3- (4,5-Dimethylthiazol-2-yl)-2,5-diphenyl-2H-tetrazolium Bromide; Methylthiazolyldiphenyl-tetrazolium bromide (MTT)] were purchased from Sigma (St. Louis, MO, United States). Propidium iodide (PI) and FITC-Annexin V were both purchased from BD Biosciences (San Jose, CA). RNA extraction kit, PrimeScript RT MasterMix kit, and SYBR Premix Ex Taq kit were provided by Takara (Dalian, China). Antibodies against POLE2 was obtained from Santa Cruz (CA, United States). Antibodies against FOXM1 and actin as well as secondary antibodies were purchased from Cell Signaling Technology (Danvers, MA, United States).

### Microarray Data Analysis

A549 cells were treated with 120uM berberine for 24 h. Total RNA of berberine treatment and non-berberine treatment were extracted using the Trizol reagent (Invitrogen, Life Technologies) and reversely transcribed to complementary DNA (cDNA) using the Quantscript RT kit (Tiangen Biotech). Hybridization was performed using the lllumina Human-12Tv4 Expression BeadChip system (lllumina, San Diego, CA), which contains 47,231 probes per array product. Slides were scanned by GeneChip® Scanner 3,000 (Cat#00-00212, Affymetrix, Santa Clara, CA, US) and Command Console Software 4.0 (Affymetrix, Santa Clara, CA, US) with default settings. Raw data were normalized by MAS 5.0 algorithm. Statistical significance of differential expression of probe sets between groups were detected by student t test. Probe set with *p*-value ≦ 0.05 and absolute fold change ≧ 1.5 were considered as differential expressed. One gene keeps only one most statistical significant differential expressed probe set.

### Functional Characterization of DEGs

The KEGG database (Release 91.0) and Gene Ontology (GO) category database were used for functional annotation of differentially expressed genes. Enrichment analysis of GO categories was performed by R clusterProfiler (v 3.14.3) package, and enrichment analysis of pathways was tested upon hypergeometric distribution by R “phyper” function. Those GO categories a FDR < 0.05 were considered as significant enriched. While pathways with a *p*-value < 0.05 were regared as enriched. Only those GO categories or pathways contains ≧ 5 DEGs were kept for further analysis.

### Gene Set Enrichment Analysis

Genes were ranked by log2 (Foldchange). Annotated gene set “c2.cp.kegg.v7.1.symbols.gmt” was selected as the reference gene set downloaded from the Molecular Signatures Database (MSigDB), and p adjust <FDR and absolute NES >= 1 was considered significant. R package “clusterProfiler” were used to do this analysis. Then we plot the Cell Cycle and DNA repliaction gene sets in the enrichment score.

### Protein–Protein Interaction

The interactions between the protein products of DEGs were collected from Pathway Commons database (http://www.pathwaycommons.org/), Pathway Commons provides directed protein-protein interactions. Two criteria were used: Number of protein–protein interactions (PPIs) and largest connected component. 1,000 randomized networks were created. Pvalue were calculated by compared the observed value with values of randomized networks.

### Reproducibility Assessment Using TCGA Dataset

The gene expression profiles data of 526 LUAD patients and 59 adjacent cancer samples and clinical characteristics of matched patients were obtained from the Cancer Genome Atlas (TCGA) data portal (Liu et al., 2018). LUAD sequencing data were downloaded. Differential expression of genes between patients and adjacent were detected by “edgeR” (v 3.28.1) package, with a threshold of a FDR ≦ 0.05. Then functional enrichment analysis were performed on these DEGs as described above. The clinical data of 522 patients were used for Cox regression analysis.

### Cell Culture and Plasmid Transfection

Human NSCLC cell lines, A549, H1299, and H1975 cells were purchased from Shanghai Cell Bank of the Chinese Academy of Sciences. A549 cells were cultured with DMEM medium and H1299 and H1975 cells were grown in RPMI 1640 medium. Both DMEM medium and RPMI 1640 medium were supplemented with 10% fetal bovine serum, penicillin (100 U/ml) and streptomycin sulfate (100 μg/ml). Cells were maintained at 37°C in a humidified 5% CO2 atmosphere. The empty plasmid EX-NEG-M02 (Genecopoeia) and human FOXM1-overexpression plasmid (Genecopoeia) were transfected into A549, H1299, and H1975 cells using Lipofectamine 3,000 reagent (Invitrogen #2024201, MA, United States), according to the manufacturer’s instructions. After that, the A549, H1299, and H1975 cells transfected with the plasmid were used in subsequent experiments.

### MTT Colorimetric Analysis for Determining Inhibition of Cell Proliferation

To assess the effect of berberine on the survival and proliferation of NSCLC cells, MTT analysis was adopted. A549, H1299 and H1975 cells in the logarithmic phase were seeded in a 96-well plate, at a density of 3,000 cells/well and maintained at 37°C in a humidified 5% CO2 atmosphere for 16 h. Then the berberine at a density of 0, 30, 60, 90, 120, 150, 180, 210, 240, 270 μM were added, respectively. The total volume was 200 μL. Every concentration was set as well with four parallel wells in each group and kept at 37°C in a humidified 5% CO2 atmosphere for 24, 48 and 72 h, respectively. When the time was due, 20 μL of MTT reagent (Sigma) was added to each well in the 96-well plate. Then the plate was kept at 37°C for 4 h. After removing the supernatants, 150 μL of DMSO was supplemented into each well and then the plate was maintained at 37°C for 15 min. Absorbance (A) was determined with the microplate reader at 570 nm. The cell inhibition was calculated as follows:

Inhibition rate (%) = [(A of negative control group - A of test group)/A of negative control group] ×100%

### Determination of Sphere Formation Efficiency

To clarify the effect of berberine on the tumorigenesis ability of NSCLC cells, plate clone assay was applied. A549, H1975 and H1299 cells (2000 for each type) in the logarithmic phase were seeded in a 6-well plate for 16 h. Then berberine at a density of 0, 5, 10 and 20 μM were added to NSCLC cells, respectively. The cells were maintained at 37°C in a humidified 5% CO2 atmosphere. Ten days later, the colonies were stained with crystal violet and photographs of the stained colonies were taken by the digital camera and dissecting microscope.

### Determination of the Effects on Transplanted Tumor in C57BL/6 Mice

C57BL/6 mice aged at 6–8 weeks and weighed at 18–22 g were purchased from SLAC Shanghai and fed in a standard feeding atmosphere at Jiangnan University. LLC cells (3 × 106) were suspended in the medium at 150 μL and then subcutaneously injected into the right axilla of C57BL/6 mice. After LLC cells were injected, berberine groups were given an intraperitoneal injection of berberine (100, 200 and 400 mg/Kg) continually for 4 weeks. The blank control group was administered intraperitoneally injected with PBS. The volume of the tumor was measured every 3 days with a vernier caliper. The formula for volume was as follows: V=(П/8) × a × b2. “a” represents the maximum diameter of the tumor, while “b” reflects the shorter diameter vertical to “a”. The C57BL/6 mice with the tumor over 2000 mm3 was put to death by CO2 suffocation.

### HE Staining of Mouse Tumor Were Observed

The tumors of mice were taken and soaked in 4% paraformaldehyde solution. The 4% paraformaldehyde solution was changed every day. One week later, the fixed specimens were routinely dehydrated with alcohol gradient, transparent xylene, embedded with paraffin, and sectioned 5 m thick. The tumor sections were stained with HE and were observed under microscope.

### Immunohistochemistry Analysis

To demonstrate the expression of FOXM, immunohistochemistry was used to detect. The tumor sections of mice in each group were incubated with FOXM1 (Cell Signaling Technology, MA, United States) and detected, respectively, with the secondary antibodies (Cell Signaling Technology, MA, United States).

### Quantitative RT-PCR

Total RNA from A549, H1299 and H1975 cells was extracted using Trizol reagent after the treatment of 0, 30, 60, 120 μM berberine for 24 h. 1 μg of total RNA was transcribed to cDNA using the PrimeScriptTM RT Master Mix kit (TaKaRa, China) aberberineording to the protocols. Quantitative RT-PCR was performed in a reaction volume of 20 μL cDNA on ABI system (Applied Biosystems, Life Technologies), and carried out with the following parameters: 95°C for 30 s, amplifications were carried out with 40 cycles at a melting temperature of 95°C for 5 s and an annealing temperature of 60°C for 30 s, followed by melt curve analysis. The relative expression was calculated using the 2−△△Ct. The following four genes were selected for analysis: RRM1, RRM2, POLE2, and LIG1. An 18 S was used as an internal reference gene to normalize the expression of all genes. Primers for all genes were listed as follows: 18 S forward: GTA​ACC​CGT​TGA​ACC​CCA​TT; 18S reverse: CCA​TCC​AAT​CGG​TAG​TAG​CG. POLD1 forward: ATC​CAG​AAC​TTC​GAC​CTT​CCG; POLD1 reverse: ACG​GCA​TTG​AGC​GTG​TAG​G. DNA2 forward: AGA​GCT​GTC​CTG​AGT​GAA​ACT; DNA2 reverse: GAA​ACA​CCT​CAT​GGA​GAA​CCG. POLE2 forward: TTT​TGC​AGA​AGT​CTT​CAC​AGA​TG; POLE2 reverse: GCA​GAA​GGT​TGG​TTT​GAA​GA. RFC5 forward: GAA​GCA​GAC​GCC​ATG​ACT​CAG; RFC5 reverse: GAC​CGA​ACC​GAA​ACC​TCG​T. MCM2 forward: ATC​TAC​GCC​AAG​GAG​AGG​GT; MCM2 reverse: GTA​ATG​GGG​ATG​CTG​CCT​GT. MCM4 forward: CTG​TCC​ATT​GCA​AAG​GCT​G; MCM4 reverse: GAG​ACT​CAA​TGG​GAT​TTG​CTG. MCM6 forward: CTG​TGA​TGA​GGT​CCA​ACC​T; MCM6 reverse: GAC​ATC​AGG​TGT​TTC​CAC​AC. RFC3 forward: CCC​TGA​GAC​AGA​TTG​GGA​G; RFC3 reverse: TCA​AGG​AGC​CTT​TGT​GGA​G. FEN1 forward: GAT​GAT​TTC​TTC​AAG​CCT​TGA​C; FEN1 reverse: TCA​CAA​ACA​CAG​ACA​CAG​C. POLA2 forward: CTC​TCC​AAG​TGC​TAC​TCC​C; POLA2 reverse: ATA​CTC​CCT​GTG​CTA​AGC​C. PRIM1 forward: CTT​AAA​CTT​TAT​TAC​CGG​AGG​C; PRIM1 reverse: GTA​ATT​CTT​TAT​CAC​TCC​ACC​G. PRIM2 forward: GGT​TTA​ACT​TTG​GAA​CAG​GC; PRIM2 reverse: TTC​CTC​TTC​CTT​GTC​TGG​A; FOXM1 forward: ATA​CGT​GGA​TTG​AGG​ACC​ACT; FOXM1 reverse: TCC​AAT​GTC​AAG​TAG​CGG​TTG.

### Western Blotting for Determining Protein

A549, H1299 and H1975 cells in the logarithmic phase were seeded in 10 cm culture dishes. When cells occupied 80% of the dish, berberine were used for intervention. berberine at a density of 0, 30, 60, 120 μM were added to A549, H1299 and H1975 cells, respectively. 24 h later, 100 μL RIPA was added to the dishes and cells were fully soaked. After centrifugation for 30 min, supernatants were collected for protein measurement. After being washed with 95°C water for 10 min, the proteins were separated with gel and transferred to a membrane. The membranes were incubated overnight at 4°C with the primary antibodies and then further incubated with secondary antibodies and finally visualized.

### Statistical Analysis

The relationship between Cell cycle or DNA repair pathway related gene expression and overall survival of TCGA—LUAD patients was analyzed using COX regression. *p* < 0.05 was considered to have significant statistical significance. all these analysis were conducted using R (v. 3.6.0).

## Results

### Altered Pathway and Genes After Berberine Treatment

**FIGURE 1:**
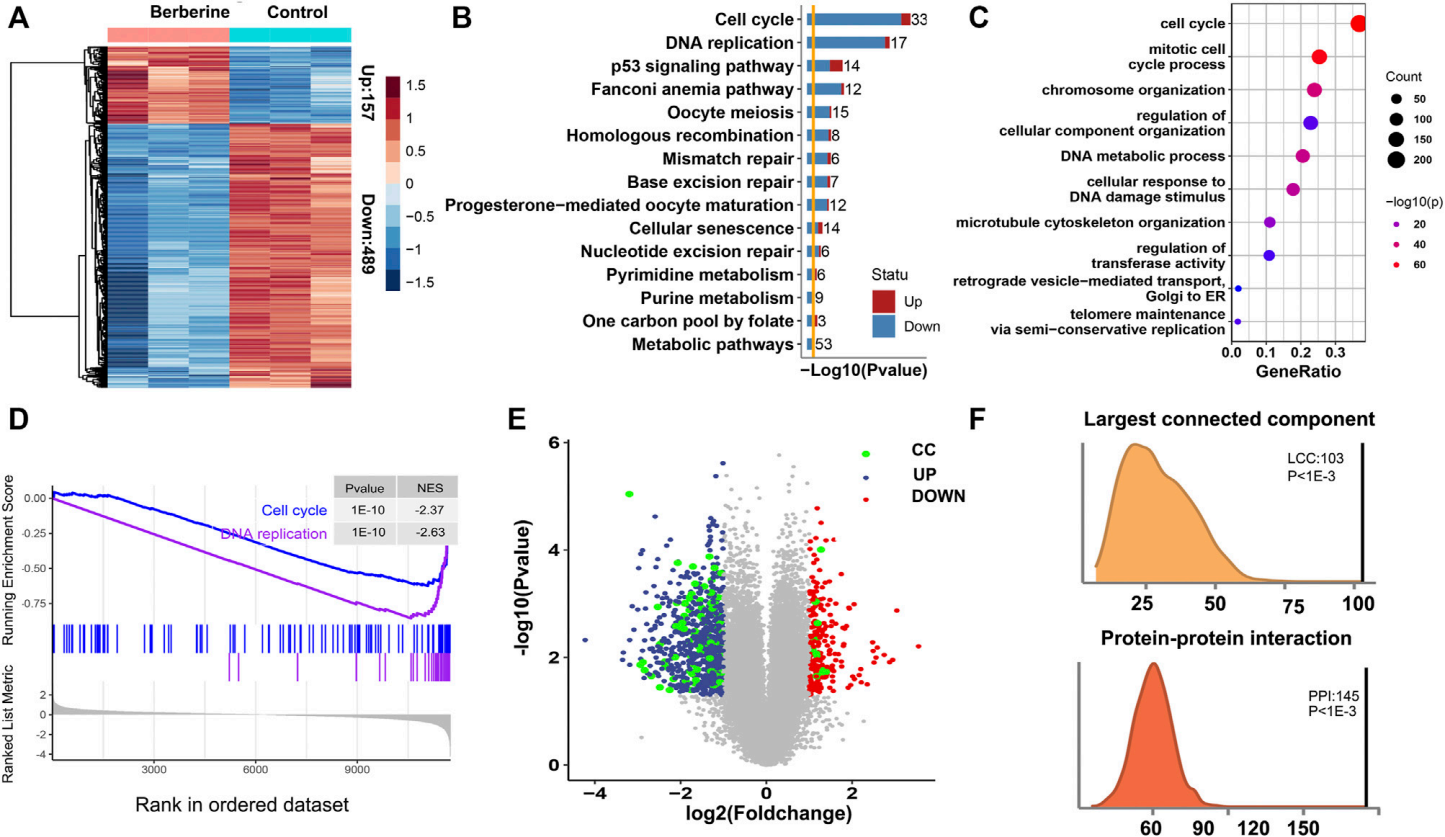
Potential antitumour mechanisms of berberine in NSCLC. **(A)** Heatmap of differential expression gene human A549 cells after berberine treatment 157 genes were up-regulated and 489 genes were down-regulated. **(B)** KEGG enrichment analysis showed that the DEGs were mainly enriched in Cell cycle and DNA replication pathway after berberine treatment, and tended to be down-regulated. **(C)** Gene Ontology enrichment analysis showed that the DEGs were mainly enriched in Cell cycle gene set. **(D)** Enrichment Score of Cell Cycle and DNA replication pathway in the Berberine cohort. **(E)** Volcano plot of DEGs. Up-regulated genes were colored in red, down-regulated genes in blue and Cell cycle and DNA replication related genes in green. Cell cycle and DNA replication related genes were significantly down regulated. **(F)** Density plot of number of protein-protein interactions (PPIs) in the 1,000 randomized network and the observed PPIs, revealing significant enrichment for PPIs between CC-DEGs and other DEGs relative to random expectation (*p* < 10^−3^); Density plot of size of the largest connected component (LCC) in the 1,000 randomized network and the observed LCC, revealing CC-DEGs densely interact with other DEGs (*p* < 10^−3^).

To screen for potential differentially expressed genes (DEGs), we analyzed the transcriptome of three berberine treated and three untreated A549 cells by microarray. The results showed that nearly 76% of DEGs were down-regulated after berberine treatment, compared with control group (Figure 1A). KEGG pathway and Gene Ontology enrichment of these DEGs after berberine treatment were highly enriched in Cell cycle and DNA replication pathway, and tended to be down-regulated (Figures 1B,C). And we reproduced this finding by Gene Set enrichment analysis (GSEA). GSEA revealed that Cell Cycle and DNA replication pathway were the most enriched (Supplementary Figure S1), and A549 cells treated with berberine showed an declining phenotype of Cell Cycle and DNA replication (Figure 1D). Therefore, we guess the DEGs of Cell Cycle and DNA replication might have crucial roles after berberine treatement. We name these DEGs as CC-DEGs and visualized in a scatter plot with the x-axis as log2 (FoldChange) and y-axis as–log10 (Pvalue). The CC-DEGs were colored in green. Some of the CC-DEGs shows high differential expssion at the down-regulated region with high–log10 (*p*-value) and absolute log2 (FoldChange) (Figure 1E). Next, we investigate the tendency for the protein products of CC-DEGs to physically interact with other DEGs. Enrichment for protein-protein interactions (PPIs) between CC-DEGs and other DEGs was observed relative to random expectation (Figure 1F, empirical *p* < 10^−3^). We then evaluated whether Cell cycle and DNA replication genes densely interact with other DEGs by calculating the size of the largest connected component between CC genes and other DEGs, and found that these genes collectively formed a significantly larger subnetwork than random expectation (Figure 1D, empirical *p* < 10^−3^). This results further indicate that the important role of CC-DEGs after berberine treatment.

### Berberine Down Regulated Survival Related Genes in Cell Cycle and DNA Replication Pathway

**FIGURE 2:**
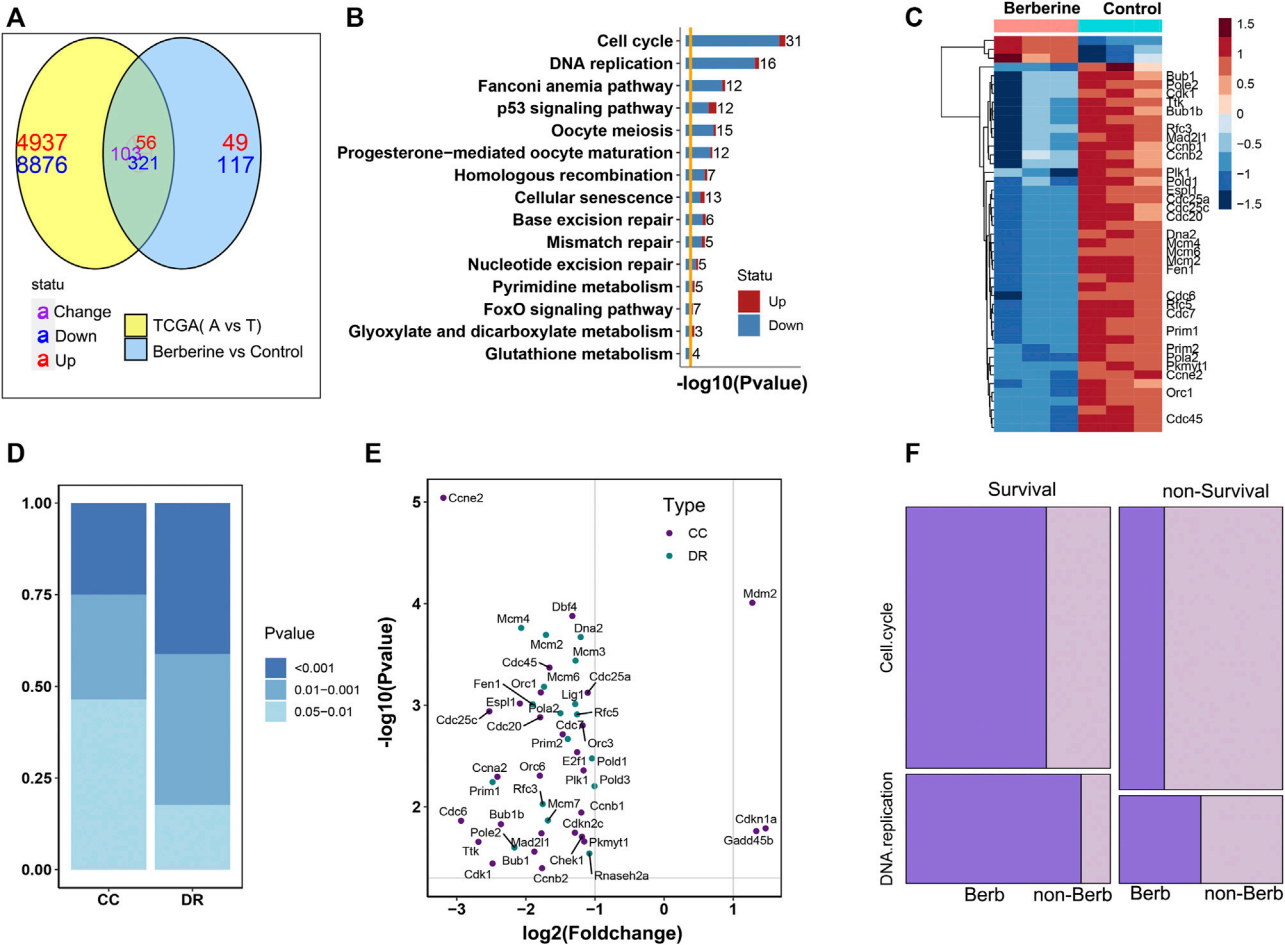
Berberine down regulates survival related genes in DNA replication pathway. **(A)** The Venn map of DEGs in berberine cohort and TCGA-LUAD cohort showed that berberine could change the up-regulated genes into down-regulated genes, and the enrichment of (321 + 56) genes with the same up and down-regulated direction after berberine treatment. **(B)** KEGG enrichment analysis showed that the common differential genes in berberine treatment and TCGA were mainly enriched in Cell cycle and DNA replication pathway, and tended to be down regulated. **(C)** Heatmap of Cell cycle and DNA replication related DEGs after the treatment of berberin (only the survival related DEGs were labled on the right). **(D)** Barplot of Cell cycle and DNA replication DEGs’ distribution on three intervals cutted by *p*-value. DNA replication genes tend to have lower *p*-value. **(E)** Scatter plot of Cell cycle and DNA replication DEGs, with the x-axis as log2 (Fold change) and y-axis as–log10 (*p*-value) in the berberine cohort. **(F)** Mosaic plot of survival and non-survival related DEGs of TCGA-cohort. Survival related DEGs of berberine cohort were colored in dark violet, while non-survival related DEGs of berberine cohort were colored in light violet. The results showed that berberine significantly affected the survival genes in Cell cycle and DNA replication, especially in DNA replication pathway.

To further explore the effect of berberine on Cell cycle and DNA replication, we analyzed the data in 59 adjacent normal tissues and 526 Lung adenocarcinoma (LUAD) tumor tissues from The Cancer Genome Atlas (TCGA). 8,876 genes were up-regulated and 4,937 genes were down-regulated when tumor compared with the adjacent normal tissues by the cutoff of a FDR < 0.05. Functional enrichment of these DEGs showed that Cell cycle pathway was statistically enriched (Supplementary Figures S2A,B) and up-regulated in tumor. In TCGA, the differential genes were mainly enriched in purine metabolism and Cell cycle, and tended to be up-regulated. But, almost all of the differentially expressed genes in DNA replication were up-regulated (Supplementary Figure S2A). From this result, we learned that Cell cycle, DNA replication pathways were significant altered and up-regulated in tumor samples. Considering whether berberine could down-regulate these Cell cycle and DNA repair related genes. We compared the DEGs from comparison of adjacent of TCGA-LUAD date and 646 DEGs from berberine treated lung adenocarcinoma cells, 480 genes were common differential expressed, among which 321 were common down and 56 common up regulated (Figure 2A). Functional enrichment of these 377 coexist DEGs with the same up-down direction showed that Cell cycle and DNA replication pathways were the most significant enriched (Figure 2B). And the heatmap plot of the differential statu of these Cell Cycle and DNA replication related genes in the berberine treated lung adenocarcinoma cells showed that except for three genes, the rest genes were down-regulated (Figure 2C). Besides, the DNA replication related genes which tended to have lower *p*-value were more significant altered than the Cell Cycle genes. Among these DNA replication related DEGs, MCM2, MCM3, MCM4 and DNA2 had lower *p*-value and POLE2 and PRIM1 had larger fold change (Figures 2D,E). All these results suggested that berberine might interfere with tumor progression mainly by down regulating gene expression of Cell cycle and DNA replication.

Next, we wondered whether berberine would selectively act on survival related genes, rather than randomly altered DEGs in Cell cycle and DNA replication screened from the clinical samples. Firstly, we classify these Cell cycle and DNA replication DEGs into survival or non-survival realated by Cox ph analyasis. Genes with a *p*-value < 0.05 were regarded as survival related. We found that survival related DEGs tend to differentially expressed after berberine treatment (Figure 2F), especially reflected in DNA replication pathway. Nearly 90% survival related DEGs in DNA replication were also differentially expressed after berberine treatment, While those DEGs not alterd by berberine tended to be survival unrelated (Figure 2F), and berberine also had more significant effect on differential survival related genes in DNA replication pathway (Figure 2D). In addition, those survival related DEGs in Cell cycle or DNA replication pathway, although there was no significant difference after berberine treatment, a large proportion tended to be down regulated (Supplementary Figures S2, S3).

### Berberine Down Regulated the Expression of FOXM1 Related to Survival

**FIGURE 3:**
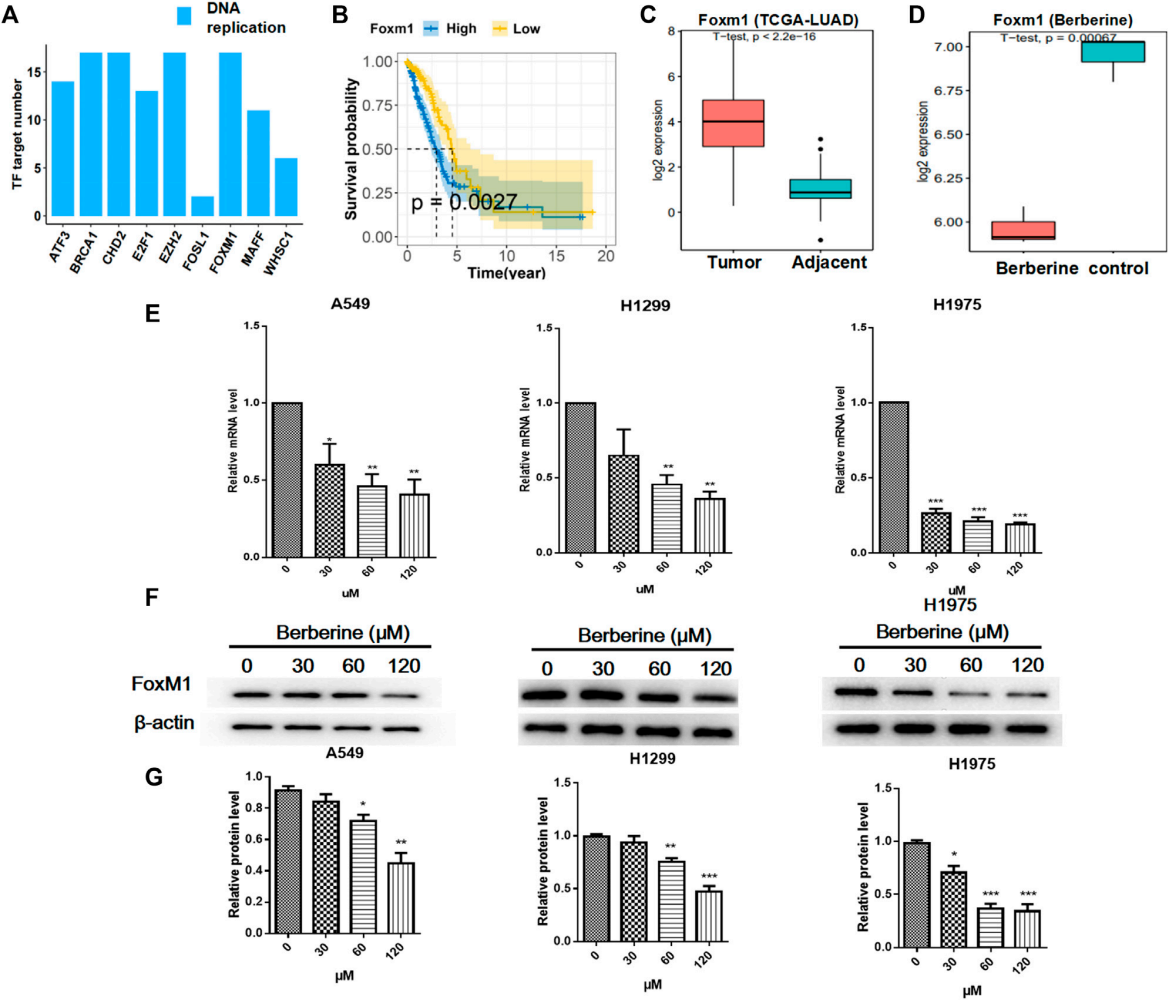
Berberine down regulated the expression of FOXM1 related to survival. **(A)** Number of target gene regulated by TF or TF cofactors. These target gene were DNA replication pathway related differential genes. **(B)** Survival analysis of overall survival in FOXM1 high and low expressed (cutted by median expression) groups of patients of TCGA-LUAD Cohort. *p* value from Kaplan-Meier analysis. **(C,D)** Boxplot of the expression level of FOXM1 in TCGA-LUAD cohort and berberine treatment cohort. *p* value from student t-test. **(E)** Berberine down-regulated FOXM1 mRNA levels in both A549, H1299 and H1975 cells. **(F)** Berberine down-regulated FOXM1 expression by WB in both A549, H1299 and H1975 cells. **(G)** Data were pooled from three independent experiments. *p < 0.05; **p < 0.01; ***p < 0.001, compared with the control group.

To further investigate the effect of berberine on survival related genes in DNA replication pathway, then we analyzed the upstream regulation of DNA replication related differential genes after berberine treatment. The results showed that except FOSL1, the numbers of target genes regulated by other transcription factors or their cofactors were almost the same (Figure 3A). Next, we carried out the survival analysis of the above transcription factors and their cofactors in TCGA-LUAD data, and only FOXM1 met the *p* value < 0.05, showed high expression and low survival (Figure 3B). In addition, we analyzed the differential expression of FOXM1 in TCGA-LUAD. The results showed that FOXM1 was highly expressed in clinical cancer tissues (Figure 3C), berberine treatment decreased significantly (Figure 3D). And the functional enrichment results of downstream regulatory genes of FOXM1 showed that Cell cycle and DNA replication pathway were significantly enriched, and mainly inclined to up-regulated genes (Supplementary Figure S4). After that, we verified it on non-small cell lung cancer cells. The results showed that the mRNA and protein lever expression of FOXM1 were down regulated in A549, H1299 and H1975 cells after berberine treatment, and it was the most significant in H1975 cells (Figures 3E–G).

### Berberine Inhibited the Survival of NSCLC Cells

**FIGURE 4:**
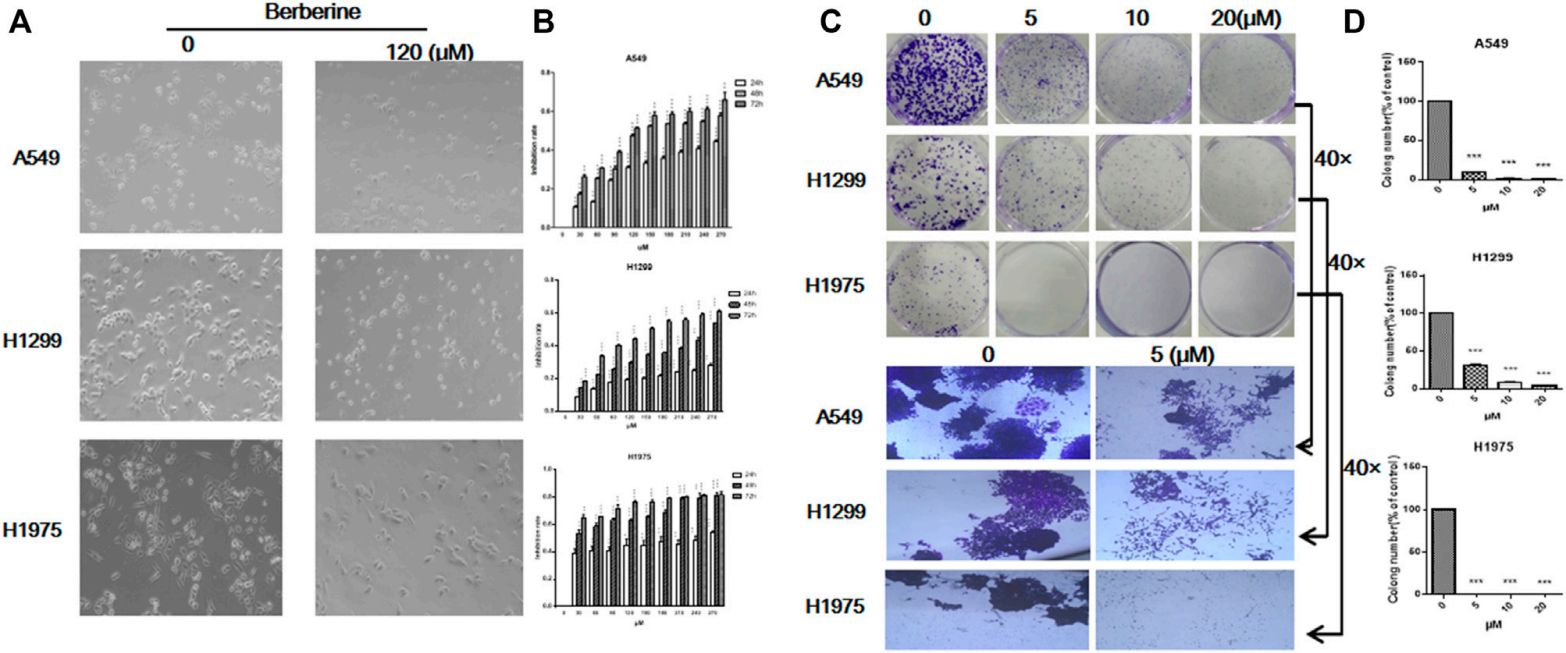
Berberine inhibited the survival of NSCLC cells. **(A)** A549, H1299, and H1975 cells were treated with berberine (120 μM) for 24 h, respectively. Photographs of cell morphology from a representative experiment are shown. **(B)** A549, H1299, and H1975 cells were treated with berberine (0, 30, 60, 90, 120, 150, 180, 210, 240, 270 μM) for 24, 48, and 72 h, respectively, and cell viability was analyzed by the MTT assay. **(C)** A549, H1299, and H1975 cells were treated with berberine (0, 5, 10 and 20 μM) and allowed to grow for an additional 10 days. Photographs of cell culture dishes from a representative experiment are shown. **(B)** Display of individual clone sizes (0 and 5 μM). **(D)** Colony percentages represent the mean ± SEM from three individual experiments. *p < 0.05; **p < 0.01; ***p < 0.001, compared with the control group.

Since berberine could inhibit the expression of FOXM1 related to survival, whether berberine could really inhibit the survival of NSCLC cells, then we carried out the cellular verification. A549, H1299 and H1975 cells were treated with berberine (0, 30, 60, 90, 120, 150, 180, 210, 240, 270 μM) for 24, 48 and 72 h, respectively. The results showed that when the concentration was 120 μM, the number of NSCLC cells was reduced and the morphology shrunked significantly (Figures 4A,B), and the inhibitory effect was enhanced with the increase of concentration (Figure 4B). Berberine is quite sensitive to H1975 cells, even at very low concentration, the inhibition rate was very high (Figure 4B). Then, we further studied the effect of berberine on the tumorigenesis of NSCLC cells by plate cloning. The results showed that after treatment of berberine (0, 5, 10, 20 μM), the clone number of NSCLC cells decreased with the increase of concentration (Figures 4C,D), 10 and 20 μM could completely inhibit the formation of NSCLC cells (*p* < 0.001, *p* < 0.001, *p* < 0.001) (Figure 4D). At high magnification, we found that the clone size of control group was very large, while that of berberine group was significantly reduced (Figure 4C). These results suggest that berberine could inhibit the survival of NSCLC cells.

### Berberine Inhibited the Survival of Lung Cancer Xenografts and Down Regulated the Expression of FOXM1 *in vivo*

**FIGURE 5:**
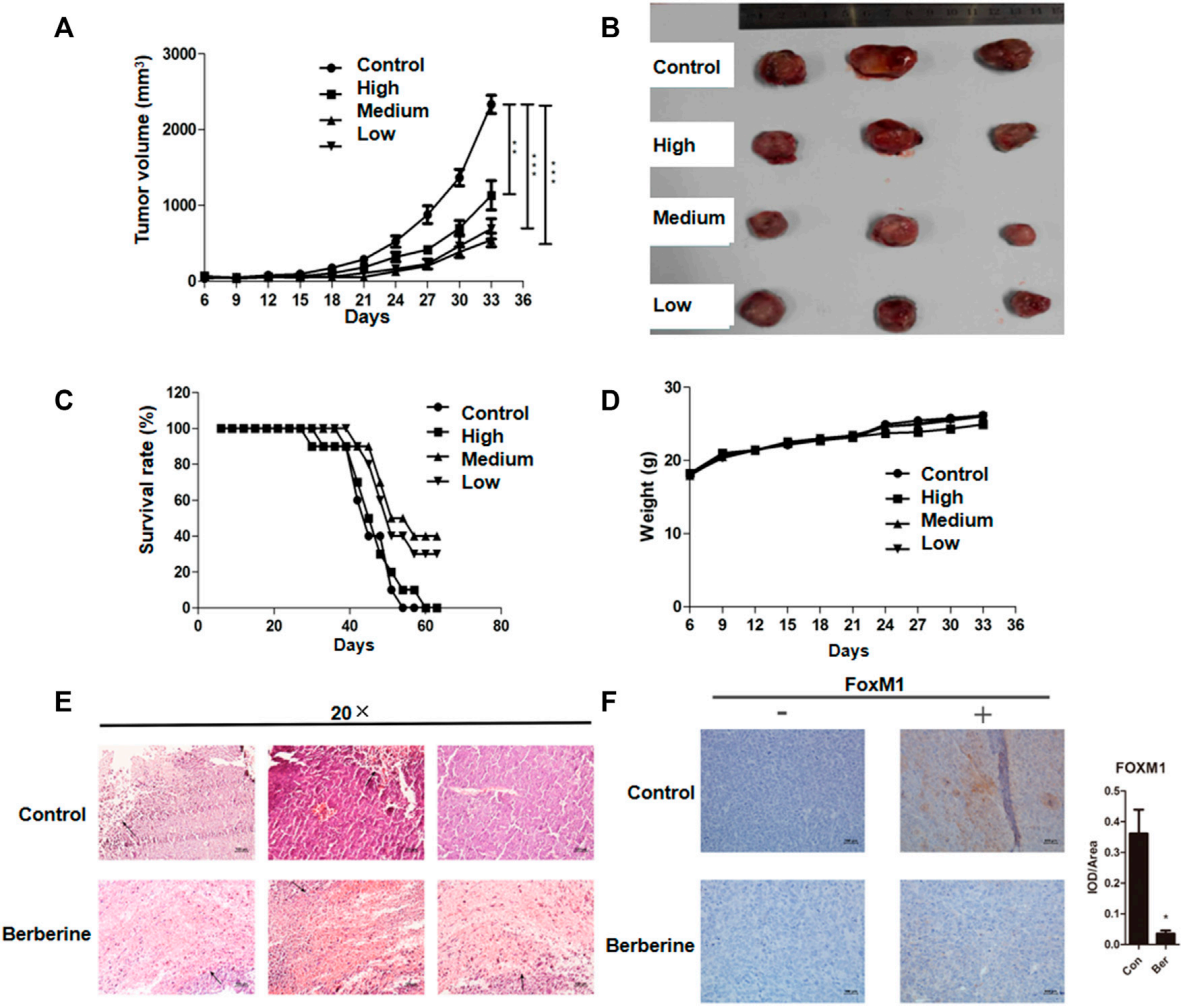
Berberine inhibited the survival of lung cancer xenografts and down regulated the expression of FOXM1 *in vivo*. **(A)** Tumor volume. **(B)** Tumor photos. **(C)** Survival. **(D)** Weight. **(E)** HE staining of the tumor (The area indicated by the arrow was the tumor necrosis area in mice.) **(F)** Compared with the blank control group, the expression levels of tumor FOXM1 in mice in the medium concentration berberine group were relatively significantly reduced (“−” refers to the absence of a corresponding antibody, while “+” refers to the corresponding antibody). Data are presented as mean ± SEM from three independent experiments. *p < 0.05; **p < 0.01; ***p < 0.001, compared with the control group.

In lung cancer cells, we have just confirmed that berberine can inhibit the survival of NSCLC cells. Then we tested the effect of berberine on the survival of lung cancer xenografts in more complex animals. We injected LLC cells into the right armpit of c57bl/6 mice. Berberine with 100, 200 and 400 mg/kg concentration were used for gastric administration. After 4 weeks, we found that high, medium and low concentrations of berberine could significantly inhibit the growth of lung cancer transplantation (*p* < 0.01, *p* < 0.001, *p* < 0.001) (Figures 5A,B), and the medium concentration of berberine had the most significant inhibition effect on the growth of lung cancer transplantation (Figures 5A,B). In addition, berberine with medium and low concentration significantly prolonged the survival time of lung cancer transplanted mice (Figure 5C), and there was no significant difference in weight between the mice in each group (Figure 5D). Then we performed HE staining and immunohistochemistry analysis of the tumor. The results of microscope showed that the tumor necrosis area of berberine group was significantly increased compared with the blank control group (Figure 5E). And berberine could significantly reduce the expression of FOXM1 in tumor (*p* < 0.05) (Figure 5F).

### Berberine Interfered the Expression of Survival Related Genes POLE2 in DNA Replication Mediated by FOXM1

**FIGURE 6:**
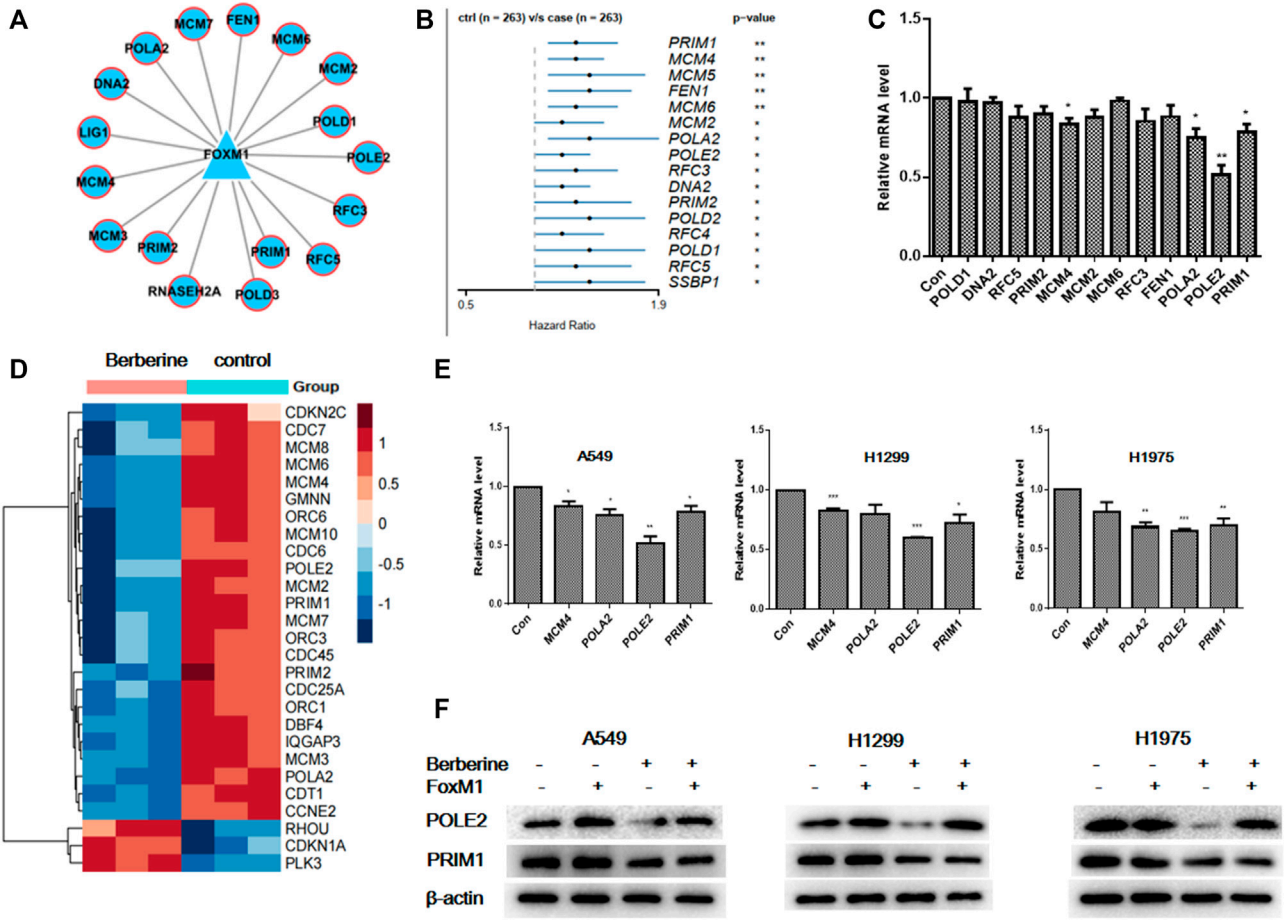
Berberine interfered the expression of survival related genes POLE2 in DNA replication mediated by FOXM1. **(A)** FOXM1 regulates DNA replication pathway related differential genes. **(B)** Forest plot of the DNA replication-DEGs RNA-expression profiles in univariate Cox proportional hazards analysis in TCGA-LUAD cohort. DNA replication-DEGs were DEGs in berberine cohort and related to DNA replication pathway. Only those genes met *p*-value < 0.05 were plotted. **(C)** We selected common genes both in DNA replication related genes regulated by FOXM1 in A549 cells and DNA replication related survival genes in TCGA, and the expression levels of them in A549 cells were tested by PCR after berberine treatment (90 μM). *p < 0.05; **p < 0.01; ***p < 0.001, compared with the control group. **(D)** The heat map showed that MCM4, POLA2, POLE2 and PRIM1 were involved in G1/S transition of mitotic Cell cycle and DNA replication, while berberine could significantly down regulate their expression, especially on POLE2 and PRIM1. **(E)** Berberine regulated MCM4, POLA2, POLE2 and PRIM1expression by PCR in both A549, H1299 and H1975 cells. **(F)** After overexpression of FOXM1 or treatment with berberine, the expression levels of POLE2 and PRIM1 were detected by WB in A549, H1299 and H1975 cells.

To verify that berberine inhibited the survival related genes in DNA replication pathway through FOXM1. We further analyzed the network relationship between FOXM1 and DNA replication related differential genes after berberine treatment, and found that not all the differentially expressed target genes were survival related (Figure 6A). Then, we used univariable Cox proportional hazards regression analysis to analyze the survival of DNA replication related genes in TCGA data, and found out the survival related genes in DNA replication (Figure 6B). We verified the common genes of the above two analyses by PCR in A549 cells and found that berberine down regulated the expression of MCM4, POLA2, POLE2 and PRIM1 in A549 cells (Figure 6C). At the same time, we found that MCM4, POLA2, POLE2 and PRIM1 were involved in G1/S transition of mitotic Cell cycle and DNA replication, while berberine could significantly down regulate their expression, especially on POLE2 and PRIM1 (Figure 6D). And berberine could significantly down regulate POLE2 and PRIM1 in A549 cells, H1299 cells and H1975 cells (Figure 6E). In addition, we found that berberine did not reduce the expression of POLE2 when FOXM1 was overexpressed, but could significantly reduce the expression of PRIM1 (Figure 6F). Moreover, we collected the peak information of FOXM1 binding sites in the pole2 genome region from the UCSC Genome Browser and found that there was a FOXM1 binding peak in the pole2 promoter region (Supplementary Figure S5). This suggested that FOXM1 bound to the POLE2 promoter region to regulate POLE2 expression. These results indicated that berberine affected the survival of NSCLC cells by inhibiting the expression of POLE2 through FOXM1.

**SCHEME 1:**
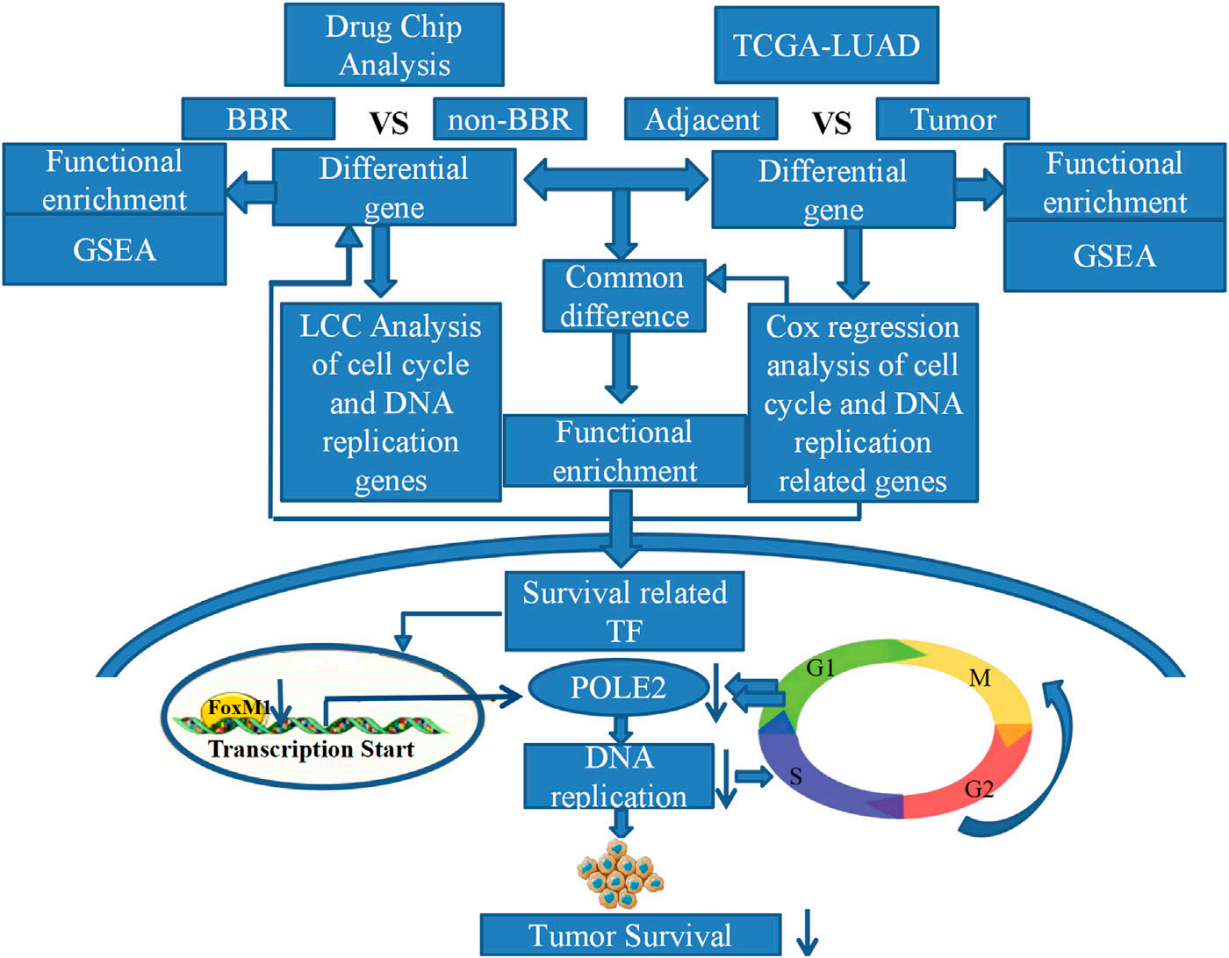
Framework diagram of the study.

## Discussion

Gene chip technology and Cancer Genome Atlas (TCGA) have been proved to be reliable diagnostic and prognostic tools for cancer patients (Sato et al., 2013; Team, 2017; Wang et al., 2017). This independent data stored in a public database enables researchers to explore the potential mechanisms of diagnosis and treatment. In this study, in order to reveal the molecular mechanism of berberine intervention in the survival of lung adenocarcinoma cells, we analyzed the differential expression of genes in A549 cells treated with berberine and in the lung adenocarcinoma cohort created by TCGA, then determined the importance of DNA replication pathway, and deeply analyzed the relationship between DNA replication of lung adenocarcinoma cells and berberine intervention in the survival of lung adenocarcinoma patients. The results showed that the differentially expressed genes were mainly enriched in the Cell cycle and DNA replication pathway after berberine treatment, and were significantly down regulated. In previous studies, we used flow cytometry to detect the cell cycle, berberine could effectively induce G1/S phase arrest of NSCLC cells. We used the samples of lung adenocarcinoma tumor and adjacent tissues in cancer genome map and lung adenocarcinoma cells before and after berberine treatment for common differential expressed gene analysis. The results showed that there were 321 + 56 common differentially expressed genes, mainly related to Cell cycle and DNA replication pathway. After berberine treatment, these differentially expressed genes were mainly downregulated, and most of these differentially expressed genes were survival related genes. Berberine had a more significant effect on differential survival related genes in DNA replication pathway than that in Cell cycle pathway. These results suggested that berberine could significantly affect the survival related genes in lung adenocarcinoma, especially in the DNA replication pathway.

It is well known that transcription factors (TF) play a major role in tumorigenesis and tumor progression by widely promoting or blocking the transcription of their targets. Identifying TF with malignant characteristics can provide a comprehensive view for explaining tumor biology. Forkhead/wing helix domain transcription factor FOXM1, as an oncoprotein, affected the occurrence and development of cancer through trans activation of related oncogenes (Halasi and Gartel, 2013; Kalathil et al., 2020). Its expression had been proved to be increased in various cancers (Halasi and Gartel, 2013; Nandi et al., 2018). It was worth noting that FOXM1 had been reported as the primary gene expression biomarker with poor prognosis in Pan-cancer analysis, which includes >18,000 tumors from 39 different malignancies (Gentles et al., 2015). The high expression of FoxM1 was closely related to the reduction of patient survival (Li et al., 2017; Barger et al., 2019). People were interested in the therapeutic target of FoxM1 in cancer (Tabatabaei Dakhili et al., 2019; Ziegler et al., 2019). FoxM1 had recently been identified as a key transcriptional regulator of related oncogenes in lung adenocarcinoma. In our study, the expression of FoxM1 was down regulated in lung adenocarcinoma cells after berberine treatment, most significantly in H1975 cells. At the same time, berberine could also inhibit the growth and clone formation of lung adenocarcinoma cells. *In vivo*, we also found that berberine inhibited the survival of lung adenocarcinoma xenografts and significantly down regulated the expression of FOXM1. In conclusion, berberine could significantly inhibit the survival of lung adenocarcinoma through FOXM1.

FOXM1 played an important role in DNA replication. FOXM1 was recently reported to induce DNA replication pressure *in vitro*, and FOXM1 expression was observed to be associated with the expression of DNA replication pressure biomarkers in several cancer types (Li et al., 2020). In order to evaluate the role of FOXM1 in DNA replication of lung adenocarcinoma cells, we further analyzed the transcriptional regulation of related differential genes in DNA replication pathway. We found that the number of target genes regulated by transcription factors or their cofactors was almost the same except FOSL1. However, in TCGA data, we analyzed the survival of the above transcription factors and their cofactors, and only FOXM1 met *p* < 0.05, showing high expression and low survival rate. We further analyzed the network relationship between FOXM1 and related differentially expressed genes in DNA replication pathway after berberine treatment, and also found that most differentially expressed target genes were related to survival. We used univariate Cox proportional hazards regression analysis to find survival related genes in DNA replication in TCGA data. Then, the common differential expressed gene in the above two analyses was verified by PCR. It was found that berberine could down regulated the expression of POLE2 and PRIM1.

It was worth noting that our study also found FOXM1 was closely related to POLE2. POLE2 was involved in cell functions, such as DNA replication, repair and Cell cycle control (Burgers, 1998), as well as array based proliferation characteristics (Rosenwald et al., 2003). On the other hand, POLE2 had been previously reported to be highly expressed in breast cancer, colorectal cancer, cervical cancer and bladder cancer (Zhou et al., 2008; Liu et al., 2014; Chubb et al., 2016). We overexpressed FOXM1 and found that berberine could not reduce the expression of POLE2, but could significantly reduce the expression of PRIM1, indicating that FOXM1 mediated the expression of POLE2. In conclusion, berberine intervened the survival of lung adenocarcinoma cells by inhibiting the expression of POLE2 mediated by FOXM1.

In conclusion, we found that berberine could significantly inhibit the DNA replication pathway in lung adenocarcinoma cells through gene chip technology of lung adenocarcinoma A549 cells and TCGA data analysis. We found that berberine could significantly inhibit the proliferation of lung adenocarcinoma cells *in vitro* and *in vivo*. In addition, we demonstrated that the therapeutic target of berberine was to inhibit the expression of FOXM1 and POLE2 mediated by FOXM1, so as to intervene the survival of lung adenocarcinoma (Overview of this study). This is the first study on the mechanism of berberine in the treatment of lung adenocarcinoma by regulating FOXM1 and POLE2 mediated by FOXM1. It is also the first time to confirm the significance and relationship of FOXM1 and its target gene POLE2 in lung adenocarcinoma.

## Data Availability

The original contributions presented in the study are included in the article/Supplementary Material, further inquiries can be directed to the corresponding authors.

## Abbreviations

Akt, Protein kinase B; Bcl-2, B-cell lymphoma-2; COX-2, Cyclooxygenase 2; ERK, extracellular regulated protein kinases; FOXM1, Forkhead Box M1; GO, Gene Ontology; LUAD, lung adenocarcinoma; miR-19a, microRNA-19a; MAPK, mitogen-activated protein kinase; NF-κB, nuclear factor-kappa B; NSCLC, Non small cell lung cancer; PRIM1, DNA Primase Subunit 1; POLE2, DNA Polymerase Epsilon 2; TCGA, The Cancer Genome Atlas; TF, Transcription factors
