# Supplementary figures and images for: Berberine Inhibits FOXM1 Dependent Transcriptional Regulation of POLE2 and Interferes With the Survival of Lung Adenocarcinoma
Source: Front Pharmacol. 2022 Jan 31;12:775514. doi: 10.3389/fphar.2021.775514 (PMC8842794; doi:10.3389/fphar.2021.775514)

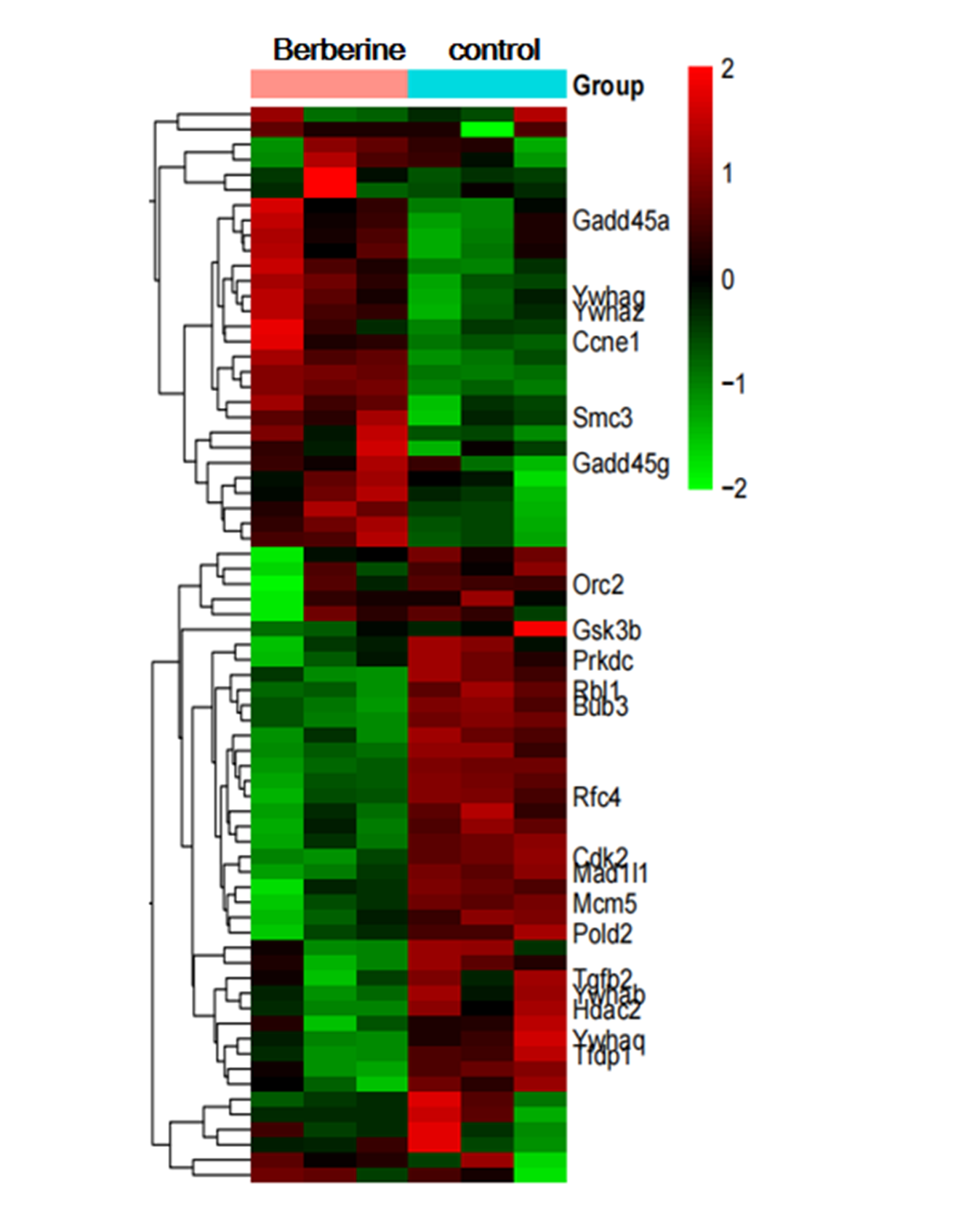

Supplement: Supplementary file 1 [file Image3.tif]

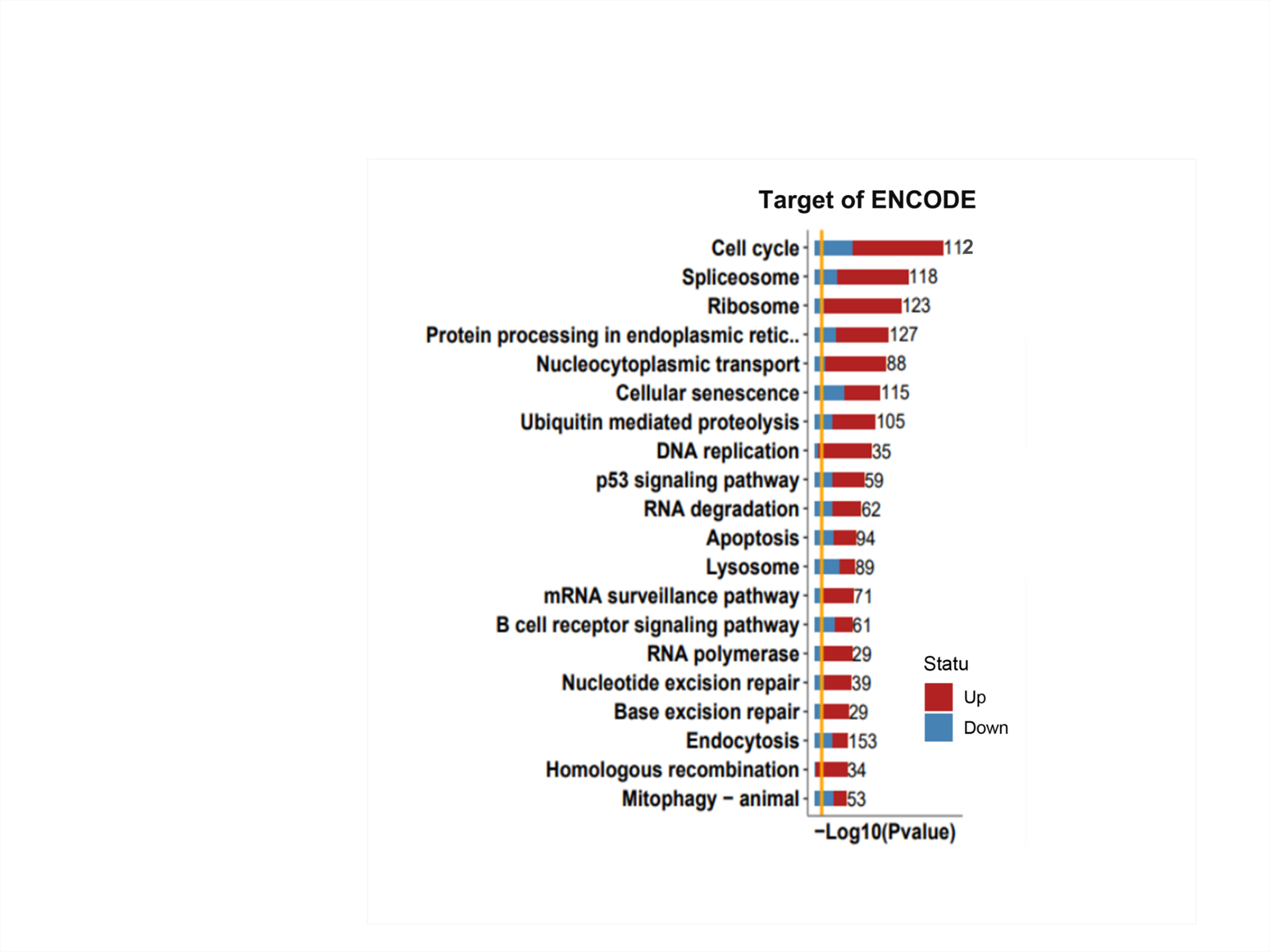

Supplement: Supplementary file 2 [file Image4.tif]

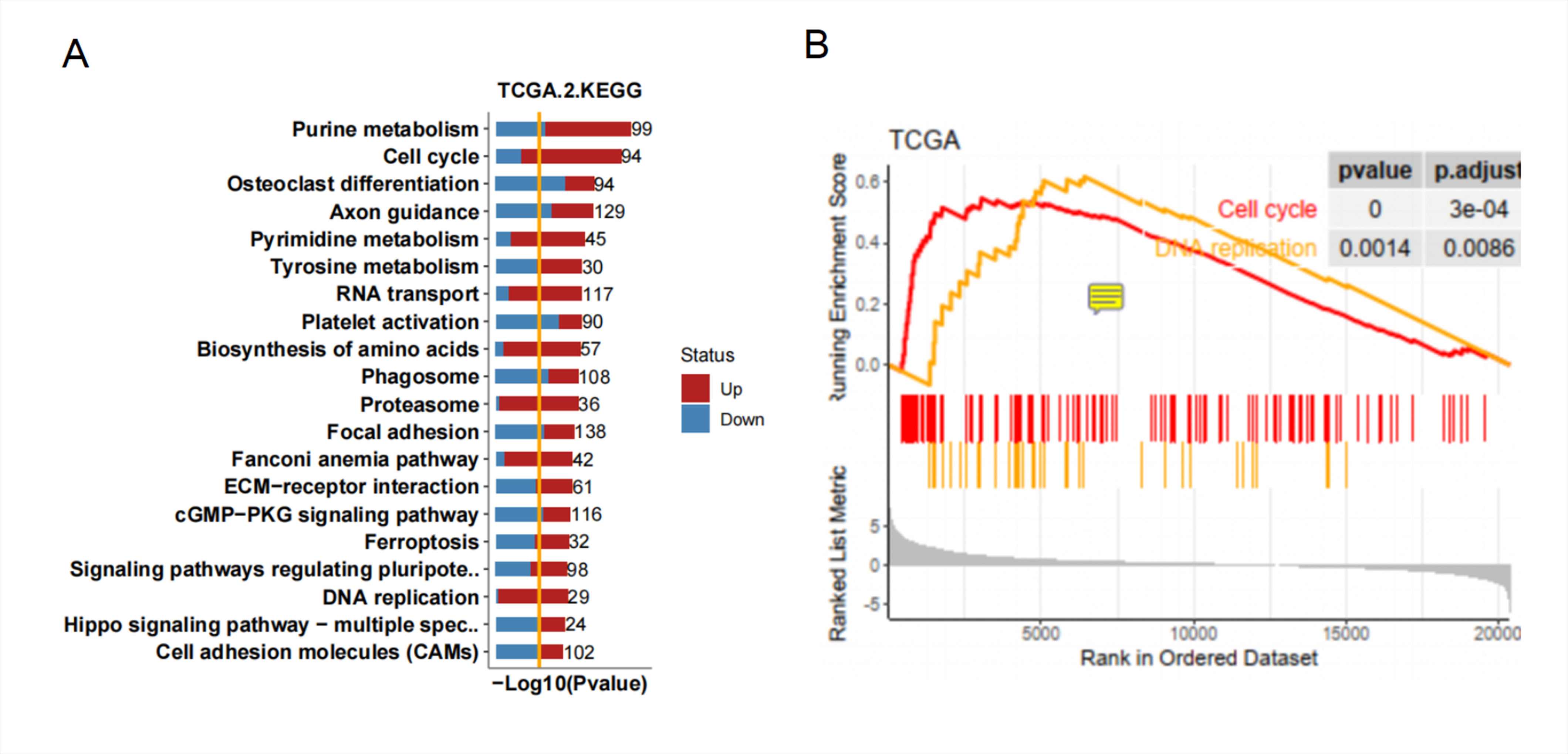

Supplement: Supplementary file 3 [file Image2.tif]

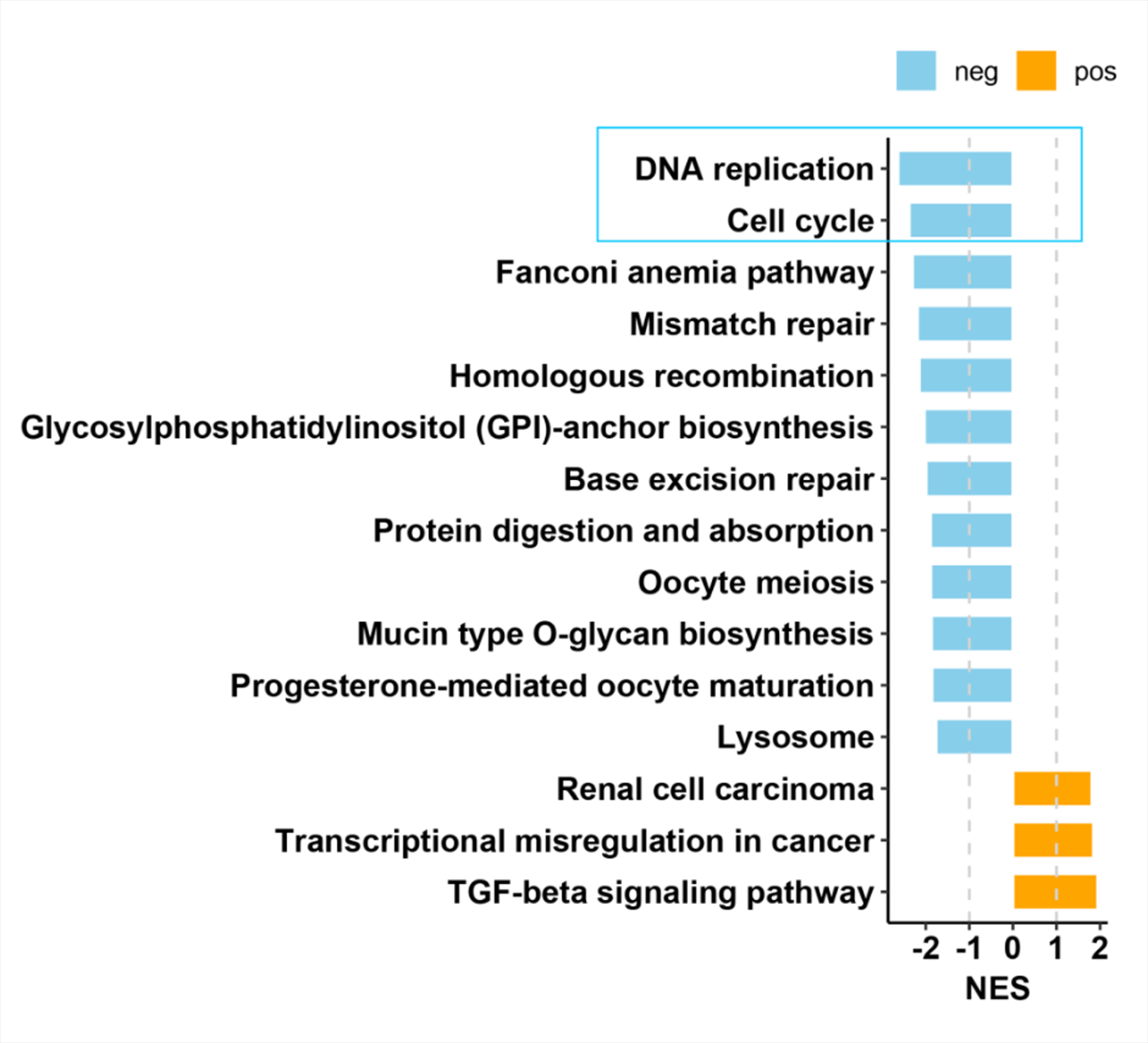

Supplement: Supplementary file 4 [file Image1.tif]

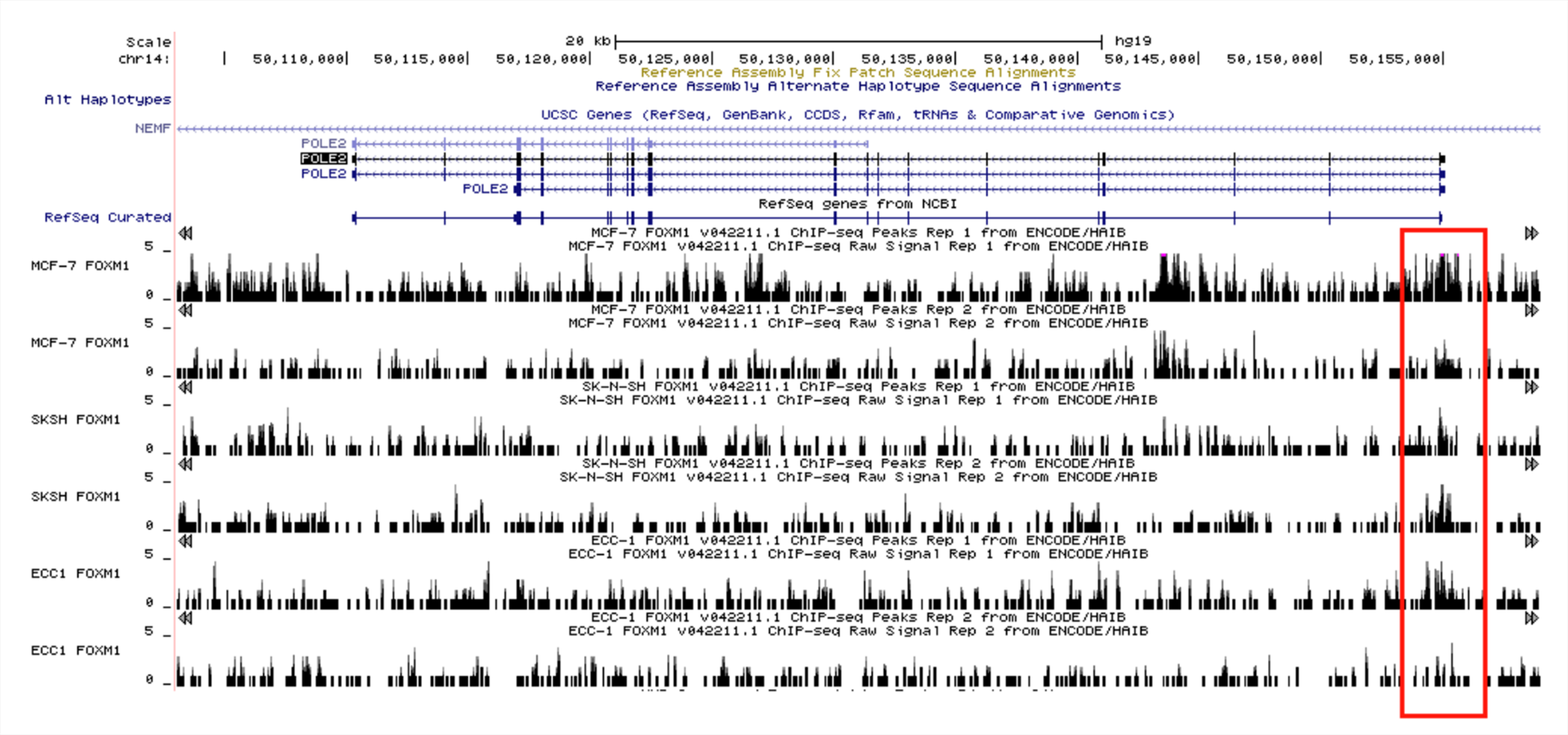

Supplement: Supplementary file 5 [file Image5.tif]
